# circRNA‐SORE/UBQLN1/GPX4 Mediates the Acquisition of Sorafenib Resistance in Hepatocellular Carcinoma Through Inhibition of Ferroptosis

**DOI:** 10.1002/mco2.70488

**Published:** 2025-11-23

**Authors:** Lin Ji, Yeling Ruan, Meng Tong, Tianyi Chen, Jingwei Cai, Zhengtao Ye, Xiujun Cai, Junjie Xu

**Affiliations:** ^1^ Department of General Surgery Sir Run‐Run Shaw Hospital Zhejiang University School of Medicine Hangzhou China; ^2^ National Engineering Research Center of Innovation and Application of Minimally Invasive Instruments Hangzhou China; ^3^ Department of Head and Neck Surgery Sir Run‐Run Shaw Hospital Zhejiang University School of Medicine Hangzhou China; ^4^ Plastic & Aesthetic Center Sir Run‐Run Shaw Hospital Zhejiang University School of Medicine Hangzhou China; ^5^ Department of Thoracic Surgery Sir Run‐Run Shaw Hospital Zhejiang University School of Medicine Hangzhou China

**Keywords:** circRNA, ferroptosis, sorafenib resistance, UBQLN1:GPX4

## Abstract

The clinical performance of targeted therapies for treating hepatocellular carcinoma (HCC) is significantly limited by the frequent emergence of drug resistance, ultimately resulting in therapeutic failure and poor prognosis. While the precise mechanisms underlying this resistance are not fully elucidated. Emerging evidence implicated that reactive oxygen species (ROS) homeostasis and ferroptosis, a unique form of programmed cell death, are closely associated with the development of drug resistance in cancer cells. In this study, we demonstrated that circRNA‐SORE, a circRNA previously reported by our group, played a crucial role in mediating sorafenib resistance via regulating intracellular ROS levels and inhibiting ferroptosis. Mechanically, we identified that circRNA‐SORE exerted its regulatory effects through modulating the level of UBQLN1. UBQLN1, via its STI domain, stabilized GPX4, a crucial antioxidant enzyme that protects against ferroptosis death. The stabilization of GPX4 promoted cancer cell survival under sorafenib‐induced oxidative stress. In conclusion, this study revealed a novel circRNA‐SORE/UBQLN1/GPX4 regulatory axis that mediated sorafenib resistance in HCC and also offered a promising therapeutic strategy to overcome drug resistance and improve clinical outcomes for patients with HCC.

## 1 Introduction

Treatment options for advanced hepatocellular carcinoma (HCC) are currently limited, highlighting an urgent need for the development of new strategies. HCC is a prevalent malignancy, ranking as the sixth most common cancer globally, and is predicted to cause one million deaths by 2030 [1]. Currently, targeted therapy and immunotherapy represent the standard therapeutic approaches for advanced HCC [2]. Guidelines from the American Society of Clinical Oncology recommend the use of atezolizumab‐bevacizumab combination therapy or tyrosine kinase inhibitors (TKIs; including sorafenib and lenvatinib) as first‐line treatments for patients with advanced HCC [3]. However, some studies have already observed the acquisition of drug resistance in HCC patients using lenvatinib [4]. This highlights the importance of studying mechanisms of drug resistance and developing strategies to overcome it in HCC. As a classic targeted agent used in the clinic for more than a decade, sorafenib has led to a high prevalence of drug resistance. Thus, it provides a useful model to study the mechanism of drug resistance, particularly for TKIs.

Several mechanisms of sorafenib resistance have been discovered. The PI3K/AKT, JAK/STAT, and hypoxia‐related pathways, as well as the epithelial‐mesenchymal transition, have been reported to contribute to the development of sorafenib resistance. However, recent studies revealed that cellular metabolism and the tumor microenvironment might contribute to the development of resistance, highlighting the need for further exploration [5]. Among them, the role of reactive oxygen species (ROS) warrants particular attention [6, 7].

Sorafenib modulates ROS levels within cells via various mechanisms. First, it inhibits tumor angiogenesis, leading to ROS generation via the hypoxia‐induced pathway. Furthermore, recent studies have revealed that sorafenib may target the mitochondrial respiratory chain, which is directly involved in the production of ROS [8]. Moreover, sorafenib was reported to promote ferroptosis efficiently through inhibiting SLC7A11 [9]. Ferroptosis can be induced by lipid peroxidation, which creates new forms of ROS [10]. These studies indicated that the cellular ROS balance, ferroptosis, and response to sorafenib are highly correlated. However, the detailed mechanism remains unclear and requires further examination.

In a previous study, we established that the circular RNA (circRNA)‐SORE plays a pivotal role in mediating sorafenib resistance by stabilizing YBX1 and activating its downstream signaling cascade [11]. Building upon these findings, the current study revealed an additional mechanistic dimension. In this study, we found that circRNA‐SORE critically modulated cellular ROS homeostasis. We observed that levels of GPX4, one of the main regulators of ferroptosis, were closely correlated with levels of circRNA‐SORE. Furthermore, dissection of the underlying mechanism uncovered UBQLN1 as a key mediator in this regulatory axis. This study thus provides a novel perspective on the molecular pathogenesis of sorafenib resistance, and suggests a promising therapeutic strategy: co‐administration of sorafenib with ferroptosis inhibitors may potentially overcome treatment resistance and improve clinical outcomes in HCC patients.

## 2 Results

### 2.1 circRNA‐SORE Affects Cellular ROS Regulation and Ferroptosis

**FIGURE 1:**
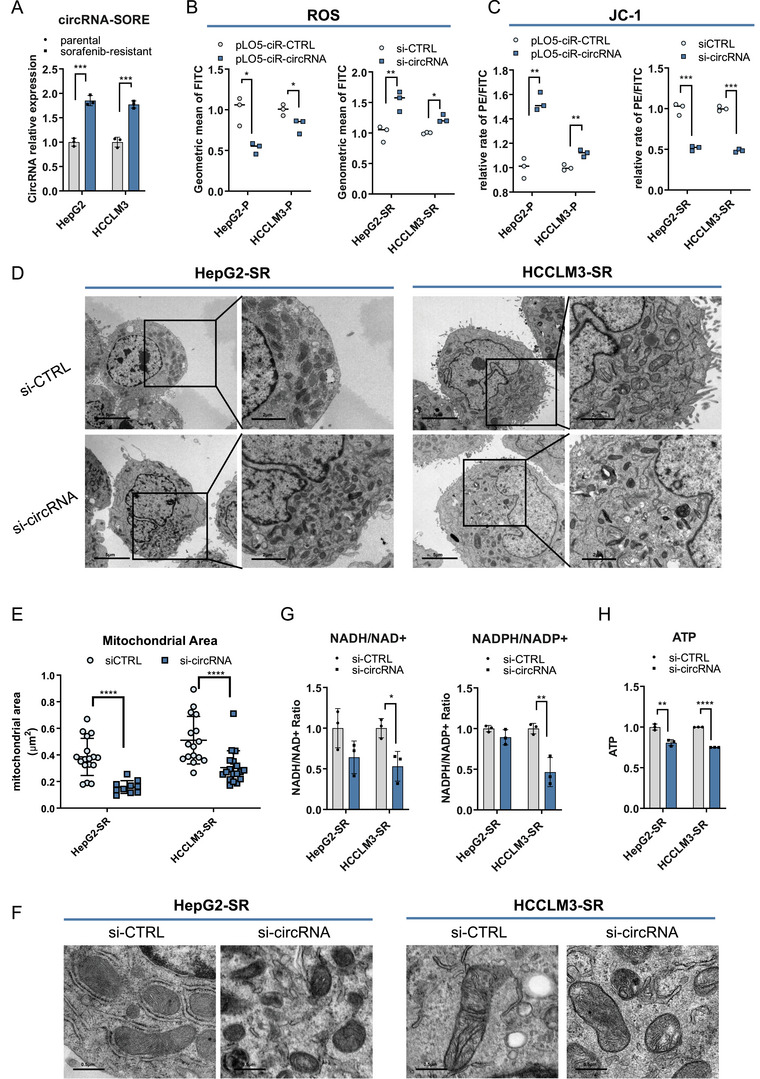
circRNA‐SORE affects cellular reactive oxygen species (ROS) regulation and ferroptosis. (A) The RNA levels of circRNA‐SORE in parental and sorafenib‐resistant cells were examined via quantitative real‐time polymerase chain reaction (qRT‐PCR) assays. (B) ROS levels were measured using a DCFH‐DA probe via flow cytometry in parental cells with or without circRNA overexpression for about 48 h. ROS levels were measured in sorafenib‐resistant cells with or without circRNA knockdown for 48 h. (C) Mitochondrial membrane potential levels were detected using the JC‐1 assay under the same conditions described in (B). (D) Representative transmission electron microscopy (TEM) images of mitochondrial morphological changes after circRNA knockdown for 48 h. Scale bar, 5 µm, 2 µm. (E). The size of mitochondria was quantified by ImageJ. (F) Representative TEM images of the changes in mitochondrial cristae after circRNA‐SORE depletion. Scale bar, 0.5 µm. (G) Cellular redox balances in sorafenib‐resistant cells with or without circRNA‐SORE depletion were monitored by detecting the level of redox pairs like NAD/NADH and NADPH/NADP. (H) ATP levels in sorafenib‐resistant cells with or without circRNA knockdown. *p < 0.05, **p < 0.01, ***p < 0.001, and ****p < 0.0001. ns. not statistically significant.

In a previous study, we demonstrated that circRNA‐SORE promotes sorafenib resistance in HCC through the stabilization of the oncogenic protein YBX1 [12]. In the current study, we investigated the effect of circRNA‐SORE on ROS regulation. Our analysis revealed significantly elevated circRNA‐SORE expression levels in sorafenib‐resistant cells (Figure 1A, Figure S1A). Notably, overexpression of circRNA‐SORE in parental cells decreased ROS levels after sorafenib treatment, while depleting it in sorafenib‐resistant cells increased ROS levels (Figure 1B, Figure S1B).

Given that mitochondria are the primary source of intracellular ROS, we then examined mitochondrial membrane integrity by measuring the mitochondrial membrane potential (MMP) and directly visualizing mitochondrial structure using transmission electron microscopy (TEM). Our results demonstrated that MMP increased significantly after circRNA‐SORE overexpression, while MMP decreased after circRNA‐SORE depletion (Figure 1C, Figure S1C). Moreover, TEM results showed that mitochondria appeared smaller and more condensed after circRNA‐SORE knockdown (Figures 1D,E, Figure S1D,E). Moreover, we observed the disappearance of mitochondrial cristae after circRNA‐SORE knockdown (Figure 1F, Figure S1F). These morphological alterations were consistent with characteristic features of ferroptosis [13].

Cellular ROS levels were closely linked to redox homeostasis. To further investigate this relationship, we quantified two key cellular redox pairs. Our data revealed significant decreases in both the NADH/NAD+ and NADPH/NADP+ ratios after circRNA‐SORE knockdown (Figure 1G, Figure S1G). Consistent with these findings, we detected reduced ATP levels after circRNA‐SORE depletion (Figure 1H, Figure S1H).

Given that GPX4 serves as the primary regulator of cellular redox homeostasis and plays a pivotal role in ferroptosis [14]. We next analyzed GPX4 expression changes during this process.

### 2.2 circRNA‐SORE May Influence Ferroptosis Through Stabilization of GPX4

**FIGURE 2:**
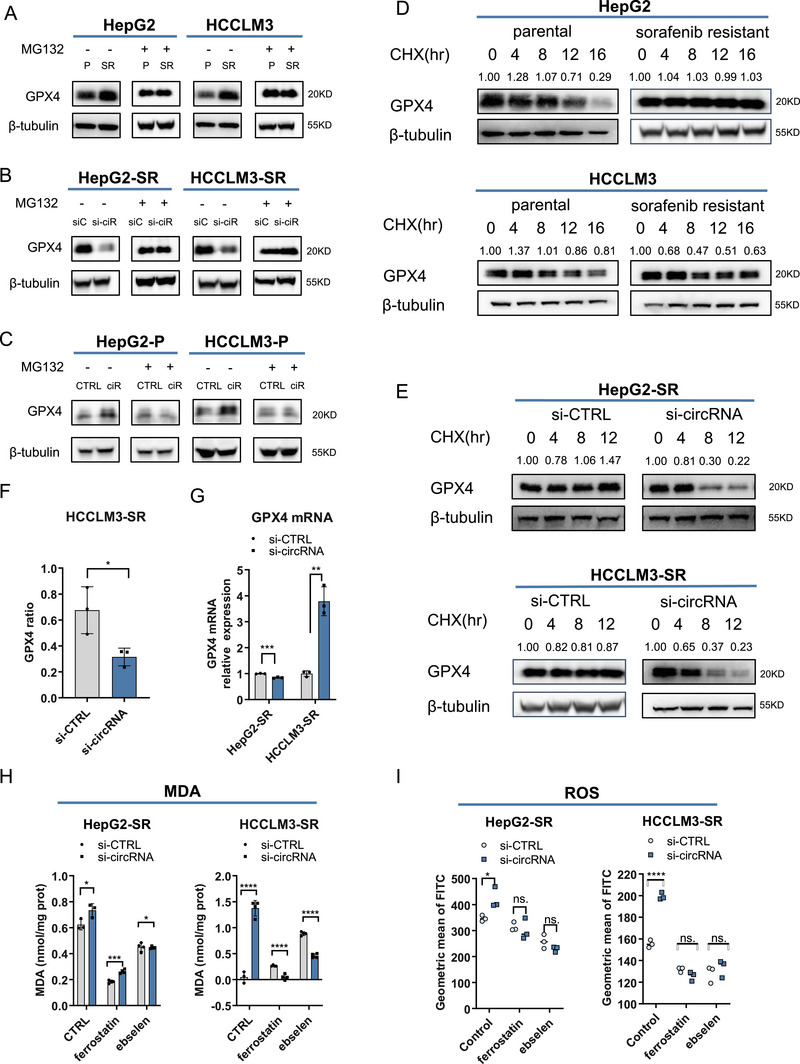
circRNA‐SORE may influence ferroptosis via stabilizing GPX4. (A) Western blot (WB) analysis of GPX4 in parental (P) and sorafenib‐resistant (SR) HepG2 and HCCLM3 cells. Cells were cultured with or without MG132 under sorafenib treatment. (B) WB analysis of GPX4 in sorafenib‐resistant HepG2 and HCCLM3 cells after circRNA depletion, treated with or without MG132. (C) WB analysis of GPX4 in parental HepG2 and HCCLM3 cells after circRNA overexpression, treated with or without MG132. (D) Degradation rates of GPX4 in parental and sorafenib‐resistant cells were measured and compared using CHX (100 µg/ml) treatment. (E) GPX4 degradation rates in sorafenib‐resistant cells with or without circRNA knockdown were analyzed through the CHX assay. (F) Results in E were quantified and analyzed using ImageJ. (G) RNA levels of GPX4 in sorafenib‐resistant cells with or without circRNA knockdown were determined by qPCR. (H) MDA analysis of cellular lipid peroxide level in sorafenib‐resistant cells with or without circRNA knockdown. Ferrostatin or ebselen was used as a ferroptosis inhibitor. (I) Reactive oxygen species (ROS) levels were measured using a DCFH‐DA probe via flow cytometry in sorafenib‐resistant cells with or without CircRNA depletion. Ferrostatin or ebselen was used as a ferroptosis inhibitor. *p < 0.05, **p < 0.01, ***p < 0.001, and ****p < 0.0001. ns. not statistically significant.

To investigate the role of GPX4 in circRNA‐SORE‐mediated sorafenib resistance, we first measured levels of GPX4 in sorafenib‐resistant and parental cells. Notably, GPX4 protein level was substantially elevated in sorafenib‐resistant cells compared with parental cells. However, this difference was attenuated by proteasome inhibitor MG132 treatment (Figure 2A). Subsequent circRNA‐SORE knockdown experiments revealed a corresponding decrease in GPX4 levels, which was similarly reversed by MG132 (Figure 2B). Similarly, overexpression of circRNA‐SORE increased GPX4 levels, while MG132 treatment attenuated this effect (Figure 2C). Besides, experiments with cycloheximide (CHX) demonstrated that GPX4 degradation in sorafenib‐resistant cells was slower compared to parental cells (Figure 2D). Accelerated GPX4 degradation was observed following circRNA‐SORE knockdown (Figure 2E,F). We further examined the messenger RNA (mRNA) level of GPX4 after circRNA knockdown. Results showed that GPX4 mRNA level slightly decreased in HepG2‐SR cells, while it significantly increased in LM3‐SR cells (Figure 2G). These transcriptional changes could not account for the consistent protein‐level alterations. Collectively, our findings indicated that in sorafenib‐resistant cells, circRNA‐SORE stabilized GPX4 protein by inhibiting its proteasomal degradation.

Given GPX4's critical role in regulating lipid peroxidation, we quantified the lipid peroxide levels by measuring malondialdehyde (MDA) alongside ROS levels. Our data demonstrated that circRNA‐SORE knockdown significantly elevated cellular MDA content. Treatment with ferroptosis inhibitors, ferrostatin, or ebselen [15] reversed this effect (Figure 2H). Consistently, the increased ROS levels induced by circRNA‐SORE knockdown were also partially attenuated by ferroptosis inhibitors (Figure 2I). Overall, these results established that circRNA‐SORE modulates both lipid peroxidation and ROS homeostasis through GPX4 regulation. Furthermore, our mechanistic studies reveal that this regulation occurs primarily through the cellular protein degradation system.

**FIGURE 3:**
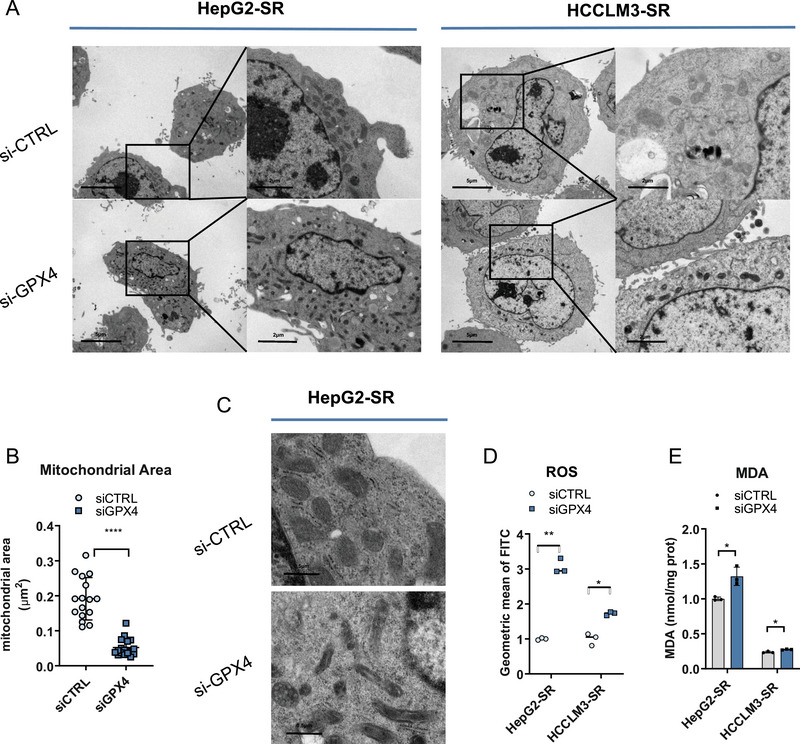
GPX4 influenced cellular reactive oxygen species (ROS) and ferroptosis. (A) Representative transmission electron microscopy (TEM) images of mitochondrial morphological changes during GPX4 knockdown. Scale bar, 5 µm, 2 µm. (B) The size of mitochondria in b was quantified by ImageJ. (C) Representative TEM images of the changes in mitochondrial cristae after GPX4 knockdown. Scale bar, 0.5 µm. (D) ROS levels were measured using a DCFH‐DA probe via flow cytometry in sorafenib‐resistant cells with or without GPX4 knockdown. (E) Cellular lipid peroxide level in sorafenib‐resistant cells with or without GPX4 knockdown was analyzed by MDA assay.

To further validate the role of GPX4 in this process, we examined mitochondrial ultrastructural changes following GPX4 knockdown through TEM. Our analysis revealed that mitochondria became smaller and more compact after GPX4 depletion (Figure 3A,B). And the damage of mitochondrial cristae was also observed (Figure 3C). We concurrently measured cellular ROS and lipid peroxide levels, confirming that GPX4 depletion significantly enhanced both ROS generation and lipid peroxidation upon sorafenib treatment (Figure 3D,E), consistent with previous reports [16]

In summary, these data demonstrated that circRNA‐SORE maintained cellular redox homeostasis and suppressed ferroptosis through GPX4 stabilization. Specifically, our results established that circRNA‐SORE exerted its protective effect by inhibiting GPX4 protein degradation, thereby preserving mitochondrial integrity and preventing lethal lipid peroxidation.

### 2.3 circRNA‐SORE Affects GPX4 Through Stabilizing UBQLN1

**FIGURE 4:**
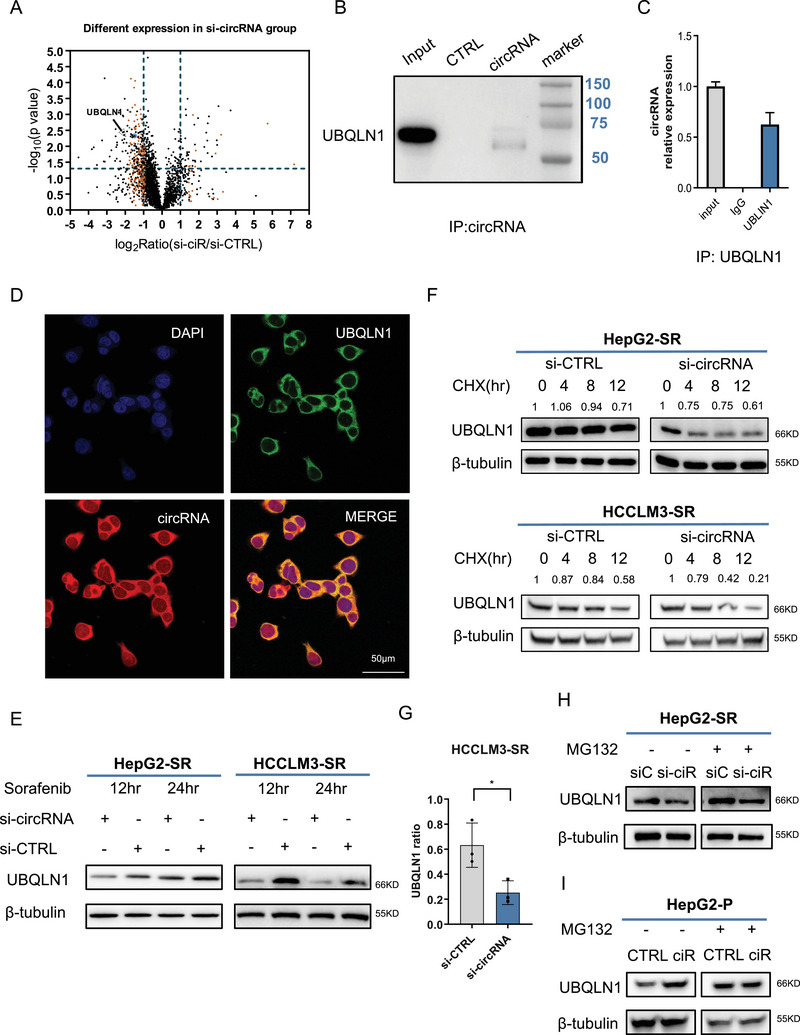
circRNA‐SORE affected UBQLN1 stability. (A) Mass spectrometry showed that UBQLN1 was reduced after circRNA knockdown. HepG2‐SR was used in the experiments. The abscissa represented the log2 value of the fold change in protein abundance. (B, C) Immunoprecipitation analysis of circRNA and UBQLN1. HepG2‐SR was used in the experiments. (D) Fluorescence confocal microscopy revealed the colocalization of UBQLN1 and circRNA. HepG2‐SR was used in the experiments. Scale bar, 50 µm. (E) WB analysis of UBQLIN1 in sorafenib‐resistant cells with or without circRNA depletion. (F) WB analysis of UBQLIN1 in cells with circRNA knockdown and sorafenib and CHX (100 µg/ml) treatment. (G) Results in F were quantified and analyzed using ImageJ. (H) WB analysis of UBQLIN1 in HepG2‐SR cells with circRNA knockdown and MG132 treatment. (I) WB analysis of UBQLIN1 in parental HepG2 cells with circRNA overexpression and MG132 treatment.

To elucidate the mechanistic basis of circRNA‐SORE‐mediated GPX4 regulation, we employed mass spectrometry to identify potential interacting partners [11]. Initial proteomic analysis revealed significant alterations in protein profiles following circRNA‐SORE depletion (Figure 4A). Another mass spectrometry was performed to identify proteins binding to circRNA‐SORE [11]. Intersectional analysis of these datasets yielded a candidate list of circRNA‐SORE‐binding proteins whose expression was circRNA‐SORE‐dependent, from which we prioritized UBQLN1 for further characterization. UBQLN1 has been reported to be a regulator of proteostasis [17] and to protect cells from oxidative stress [18]. Besides, UBQLN1 was predicted to interact with GPX3, a paralog of GPX4. RNA pull‐down assays and ribonucleoprotein immunoprecipitation (RIP) analysis were conducted to validate the physical binding between circRNA‐SORE and UBQLN1 (Figure 4B,C). Fluorescence co‐localization microscopy demonstrated that circRNA‐SORE and UBQLN1 were both localized in the cytoplasm (Figure 4D). These data indicated that circRNA‐SORE could bind to UBQLN1 physically. Functional studies revealed that circRNA‐SORE knockdown reduced UBQLN1 protein levels (Figure 4E) and accelerated its degradation (Figure 4F,G). Conversely, MG132 treatment attenuated both the circRNA‐SORE‐knockdown‐induced UBQLN1 reduction (Figure 4H) and the circRNA‐SORE‐overexpression‐induced UBQLN1 elevation (Figure 4I). From these results, we hypothesized that circRNA‐SORE stabilizes UBQLN1 through direct binding, thereby inhibiting its proteasomal degradation.

**FIGURE 5:**
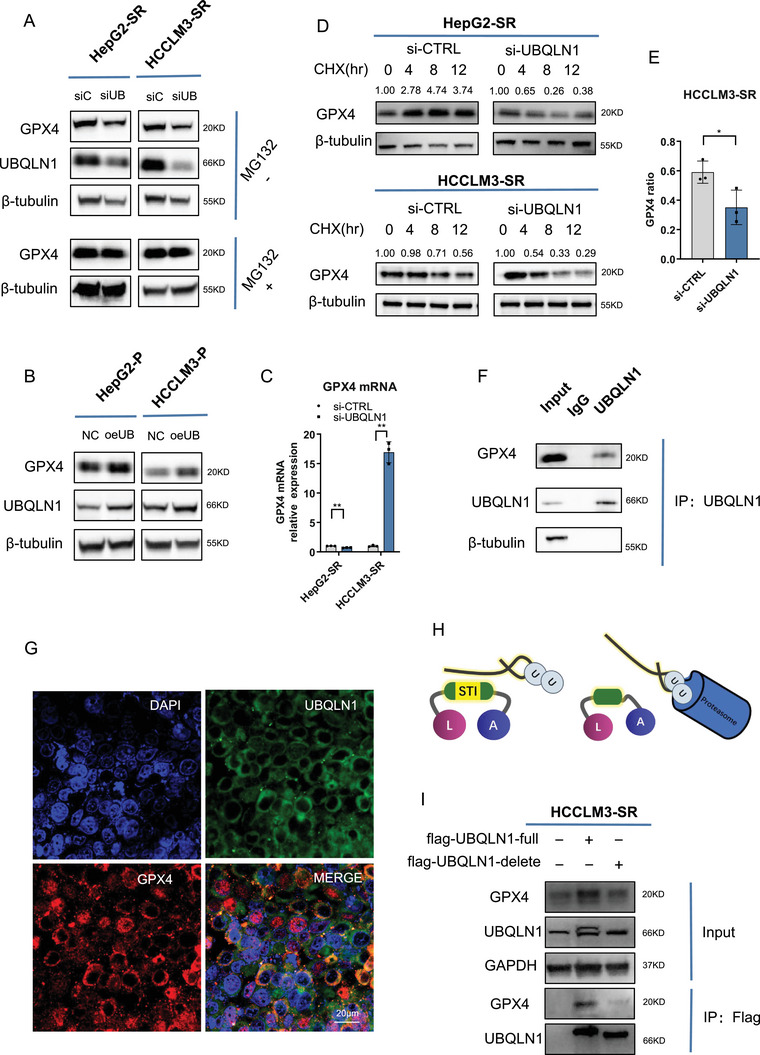
UBQLN1 affected GPX4 stability via STI. (A) Western blot (WB) analysis of GPX4 in sorafenib‐resistant cells with or without UBQLIN1 knockdown under MG132 treatment. (B) WB analysis of GPX4 in sorafenib‐resistant cells with or without UBQLIN1 overexpression. (C) Quantitative real‐time polymerase chain reaction (qRT‐PCR) assays determined the messenger RNA (mRNA) levels of GPX4 in sorafenib‐resistant cells, with or without UBQLN1 knockdown. (D) WB analysis of GPX4 in sorafenib‐resistant cells with or without UBQLN1 knockdown under sorafenib and CHX (100 µg/ml) treatment. (E) Results in D were quantified and analyzed using ImageJ. (F) Immunoprecipitation and WB analysis of GPX4 and UBQLN1. HepG2‐SR was used in the experiments. (G) Fluorescence confocal microscopy revealed the colocalization of UBQLN1 and GPX4. HepG2‐SR was used in the experiments. Scale bar, 20 µm. (H) a schematic representation of the structure of UBQLN1. (I) WB analysis of GPX4 in cells with flag‐UBQLIN1‐full or flag‐UBQLN1‐delete overexpression. Immunoprecipitation using the flag was performed. HCCLM3‐SR were used in this experiment.

To determine whether UBQLN1 directly regulates GPX4, we performed further functional experiments. Depletion of UBQLN1 significantly reduced GPX4 protein levels (Figure 5A), whereas UBQLN1 overexpression elevated GPX4 protein levels (Figure 5B). Those effects could be rescued by proteasome inhibition with MG132 (Figure 5A,B). Besides, GPX4 mRNA level alternation was not consistent with protein change (Figure 5C), and we determined that the protein alteration was not mediated by transcriptional regulation. We thus measured GPX4 degradation kinetics using the CHX assay. GPX4 exhibited accelerated degradation upon UBQLN1 depletion (Figure 5D,E). These results demonstrate that UBQLN1 stabilizes GPX4 protein by attenuating its proteasomal degradation.

Co‐immunoprecipitation assays confirmed a direct physical interaction between UBQLN1 and GPX4 (Figure 5F). Fluorescence microscopy also demonstrated that GPX4 and UBQLN1 were partially co‐localized in the cytoplasm (Figure 5G). Structurally, UBQLN1 contains three functionally distinct domains, playing critical roles in determining protein fate [19]. UBQLN1 mediates substrate recognition through its STI domain and promotes protein stabilization via its UBA domain (Figure 5H). To test the requirement of UBQLN1's STI domain for GPX4 interaction, we generated an STI domain‐deletion mutant (denoted as UBQLN1‐delete). In contrast with full‐length UBQLN1, the UBQLN1‐delete protein did not bind to GPX4 and was unable to stabilize GPX4 (Figure 5I).

Collectively, our findings established a novel regulatory axis wherein circRNA‐SORE sustained GPX4 protein level through stabilizing UBQLN1. Crucially, we demonstrated that the interaction between UBQLN1 and GPX4 depends on the STI domain of UBQLN1.

### 2.4 UBQLN1 Affected Cellular ROS Regulation and Ferroptosis

**FIGURE 6:**
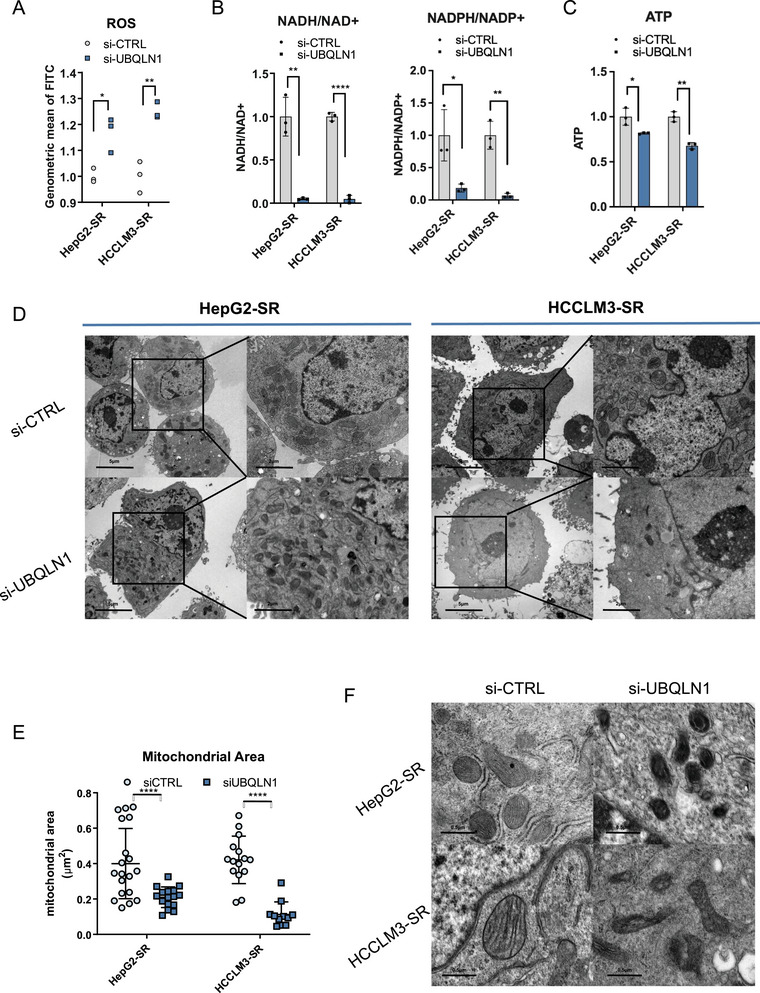
UBQLN1 affected cellular reactive oxygen species (ROS) regulation and ferroptosis. (A) ROS levels were measured using a DCFH‐DA probe by flow cytometry with or without UBQLIN1 knockdown in sorafenib‐resistant cells treated with sorafenib. (B) Redox balances between biological redox couples of NAD/NADH and NADPH/NADP were measured in sorafenib‐resistant cells with or without UBQLIN1 knockdown. (C) ATP was monitored in sorafenib‐resistant cells with or without UBQLIN1 knockdown. (D) Representative transmission electron microscopy (TEM) images of mitochondrial morphological changes during UBQLN1 knockdown. Scale bar, 5 µm, 2 µm. (E) Mitochondrial area was measured by ImageJ. (F) Representative TEM images of the changes in mitochondrial cristae after UBQLN1 knockdown. Scale bar, 0.5 µm.

To define UBQLN1's functional role in redox homeostasis and ferroptosis regulation, we performed comprehensive phenotypic characterization following UBQLN1 depletion. Our analyses revealed that depletion of UBQLN1 resulted in significantly elevated ROS levels (Figure 6A, Figure S2A), decreased NADH/NAD+ and NADPH/NADP+ ratios (Figure 6B), and decreased ATP levels (Figure 6C). Notably, TEM unveiled that mitochondria became smaller (Figure 6D,E, Figure S2B,C) and cristae disappeared (Figure 6F). Those morphological and metabolic perturbations precisely phenocopied the effects observed after GPX4 (Figure 3) or circRNA‐SORE (Figure 1) ablation. These results indicated that UBQLN1 contributed to cellular ROS regulation and inhibited ferroptosis under sorafenib treatment.

### 2.5 circSORE‐UBQLN1‐GPX4 Axis Affected Sorafenib Resistance in Vivo

**FIGURE 7:**
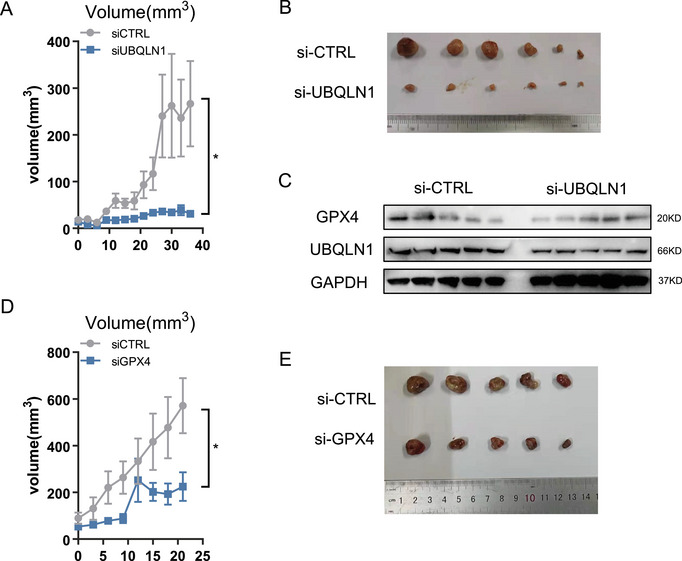
circSORE‐UBQLN1‐GPX4 axis affected sorafenib resistance in vivo. Tumor volumes were measured every 3 days after UBQLN1 siRNA injection. (B) gross representation of tumors. (C) Western blot analysis of UBQLN1 and GPX4 in tumors. (D) Tumor volumes were measured every 3 days after GPX4 siRNA injection. (E) gross illustration of tumors. *p < 0.05 and **p < 0.01. NS not statistically significant.

To validate our findings in vivo, we performed two animal experiments. Sorafenib‐resistant patient‐derived tumor xenograft (PDX) model was established through consistent sorafenib treatment for more than 4 weeks, as described previously [11]. UBQLN1 knockdown was performed through small‐interfering RNAs (siRNA) peritumoral injection. Tumor volumes were measured every 3 days. Tumors were harvested and analyzed after 36 days following injection. We found that peritumoral siRNA‐mediated UBQLN1 knockdown significantly attenuated tumor growth (Figure 7A,B), with western blot confirmation of successful UBQLN1 reduction and concomitant GPX4 downregulation (Figure 7C). These results suggested that UBQLN1 depletion partially reverses sorafenib resistance through GPX4 regulation.

The second PDX model was established similarly. In this experiment, GPX4 was depleted by siRNA peritumoral injection. Consistently, results showed that GPX4 knockdown potently enhanced sorafenib efficacy (Figure 7D,E).

In summary, these in vivo experiment results showed that knockdown of either UBQLN1 or GPX4 could enhance sorafenib efficacy. UBQLN1 exerted its function by influencing GPX4.

## 3 Discussion

While the prognosis of HCC has improved significantly in recent years, primarily due to the development of systemic therapeutic drugs, such as TKIs (sorafenib, lenvatinib, and regorafenib), their clinical performance is limited by the rapid development of drug resistance [19]. It is critical to explore the mechanisms of drug resistance in order to enhance drug efficacy and ultimately improve the prognosis. Our previous work identified the circRNA‐SORE as a key mediator of sorafenib resistance. The study demonstrated that circRNA‐SORE exerted its effect by stabilizing YBX1. In the current study, we discovered that circRNA‐SORE additionally regulated cellular ROS homeostasis, which is intimately linked to sorafenib response. Specifically, we found that circRNA‐SORE modulated redox balance and maintained mitochondrial integrity, with its depletion triggering characteristic morphological alterations, including cristae disruption. Mechanistically, we demonstrated that circRNA‐SORE maintained GPX4 protein levels, a master regulator of both ROS balance and mitochondrial function. Therefore, we proposed that circRNA‐SORE exerts its effect by stabilizing GPX4. To further investigate the detailed mechanism, we introduced mass spectrometry to interrogate proteins that interact with circRNA‐SORE. Ultimately, we identified UBQLN1 as a direct binding partner of circRNA‐SORE that is stabilized by this interaction. Further structural and functional studies revealed that UBQLN1 in turn regulates GPX4 stability through its STI domain.

Ferroptosis is a type of programmed cell death and has been recently recognized as a key tumor suppression mechanism [20]. Growing evidence suggests that ferroptosis‐targeting agents can synergize with chemotherapy, radiotherapy, and immunotherapy across multiple cancer types [9]. In HCC, sorafenib—a frontline TKI—exerts its therapeutic effects partially through ferroptosis induction [21], though its complete mechanism remains elusive. Several studies also showed that ferroptosis modulation significantly impacts sorafenib efficacy. For example, metallothionein‐1G was reported to facilitate sorafenib resistance through the inhibition of ferroptosis [22]. Conversely, the protein ACSL4 was reported to increase sensitivity to sorafenib through the promotion of ferroptosis [23]. GPX4 plays a crucial role in ferroptosis [24]. It acts as an antioxidant enzyme to protect cells from oxidative damage. Thus, GPX4 is a major regulator of tumor cell death due to ferroptosis [25]. While GPX4's role in ferroptosis is well‐established, its specific involvement in sorafenib resistance has remained unclear. Our study now demonstrates that GPX4 upregulation resulted in sorafenib resistance, aligning with prior observations in other contexts. This positions GPX4 as a promising therapeutic target for overcoming resistance in HCC patients.

Prior studies established that GPX4 is degraded by the ubiquitin‐proteasome system [26]. Increased ubiquitination of GPX4 results in the induction of both ferroptosis and apoptosis in triple‐negative breast cancer [27]. Our work now identifies UBQLN1, which was stabilized by circRNA‐SORE, as a critical guardian of GPX4 that shields it from ubiquitin‐proteasome‐mediated degradation, thereby suppressing ferroptosis in HCC.

Our study elucidates a novel molecular mechanism underlying sorafenib resistance in HCC. We revealed that circRNA‐SORE could mediate sorafenib resistance through stabilizing UBQLN1, which could stabilize GPX4 through its STI domain. GPX4 could mediate sorafenib resistance through its influence on cellular ROS regulation and ferroptosis. Furthermore, the combination of an inhibitor of the circRNA‐SORE/UBQLN1/GPX4 pathway could improve the efficacy of sorafenib treatment in HCC patients.

## 4 Materials and Methods

### 4.1 Cell Lines and Cell Culture

In this study, three human HCC cell lines, HepG2, HCC‐LM3, and SK‐Hep‐1, were obtained from the American Type Culture Collection (ATCC, Manassas, VA, USA). Cell culture procedures adhered to the supplier's instructions. Both cell lines were cultured in Dulbecco's Modified Eagle Medium (DMEM) supplemented with 10% fetal bovine serum (FBS) and maintained at 37°C in a humidified incubator with 5% CO_2_.

To generate sorafenib‐resistant variants, parental cells were gradually exposed to increasing concentrations of sorafenib over 6 months, following protocols established in previous research [28]. Cell lines that demonstrated an enhanced capacity to survive higher doses of sorafenib compared to their original counterparts were classified as resistant. These sorafenib‐resistant cells were routinely cultured in medium containing 5 µM sorafenib to preserve their resistant phenotype.

Sorafenib (Cat HY‐10201), MG132 (HY‐13259), and cycloheximide (CHX) (HY‐12320) were purchased from MedChemExpress (USA).

### 4.2 Quantitative Real‐time Polymerase Chain Reaction

Following the manufacturer's protocol, total RNA was isolated from cells using either TRIzol reagent (Invitrogen, USA, Cat. No. 15596018) or the RNA‐Quick purification kit (Esscience, China, RN001). Approximately 1 µg of this RNA was then reverse‐transcribed into complementary DNA (cDNA) with the Hifair II first Strand cDNA Synthesis SuperMix, which includes a genomic DNA digestion step (Yeasen Biotech, China, Cat. No. 11120ES60).

Quantitative real‐time polymerase chain reaction (qRT‐PCR) analysis was conducted on a Roche LightCycler 480 system (Roche Applied Science, Germany) using the Hieff UNICON qPCR SYBR Green Master Mix (antibody‐based, no Rox) (Yeasen Biotech, China, Cat. No. 11201ES03). Relative expression levels of mRNA were determined employing the ΔCt calculation method. A detailed list of primers utilized in this investigation is available in the Supporting Information.

### 4.3 Western Blotting Analysis

Cells were disrupted using RIPA buffer (Beyotime Biotechnology, China; Cat. No. P0013B). Proteins obtained from the lysates were separated through polyacrylamide gel electrophoresis and subsequently transferred onto a polyvinylidene fluoride membrane (Millipore, USA; Cat. No. IPVH00010). To prevent nonspecific binding, membranes were blocked at room temperature for 1 h with either 5% skim milk powder (Beyotime Biotechnology, China; Cat. No. P0216) or 5% bovine serum albumin (Beyotime Biotechnology, China; Cat. No. ST023). Afterwards, the membranes were incubated overnight at 4°C with the relevant primary antibody. Antigen‐antibody complexes were visualized by applying enhanced chemiluminescence detection reagents (FDbio Science, China; Cat. No. FD8030).

### 4.4 Oligonucleotide Transfection

siRNAs specifically targeting circRNA_104797, UBQLN1, GPX4, along with corresponding negative controls (siCircRNA, siUBQLN1, siGPX4, and siCTRL), were obtained from Ribobio (Guangzhou, China). Transient transfection of siRNAs into HCC cells was performed at a final concentration of 50 nM, following the manufacturer's instructions, using Lipofectamine 3000 reagent (Invitrogen, USA; Cat. No. L3000015). Gene knockdown efficiency was assessed by qPCR and Western blot analysis 48 to 72 h post‐transfection. Detailed siRNA sequences are provided in the Supporting Information.

### 4.5 Gene Overexpression

The coding sequence (CDS) of UBQLN1 was amplified from cDNA using Phanta Master Mix (Vazyme, China; Cat. No. P510‐02). The UBQLN1 deletion variant (UBQLN1‐delete) was synthesized by TSINGKE Biological Technology (China). Both sequences were cloned into the PCDH and pXF6F plasmids utilizing the ClonExpress II one‐step cloning kit (Vazyme, China; Cat. No. C112). Lentiviral particles were generated by co‐transfecting the transfer plasmid PCDH along with the packaging plasmids pMD2.G and psPAX2 (both from Addgene, UK) into suitable producer cells. Virus‐containing supernatants were harvested 48 h post‐transfection and subsequently used to infect target cells for 24 h. Following infection, cells were selected with 1 mg/mL puromycin after an additional 24‐h incubation. In contrast, plasmids constructed with pXF6F were introduced into cells via transient transfection as described previously. Additionally, circRNA‐SORE was cloned into the pLO5‐ciR vector (Geneseed Biotech, China) following the manufacturer's instructions.

### 4.6 Flow Cytometry

Intracellular ROS levels were quantified by flow cytometry utilizing the DCFH‐DA probe (Sigma, USA; CAS 4091‐99‐0). Following the respective treatments, cells were harvested and washed with phosphate‐buffered saline (PBS). The cell suspension was then incubated with 10 µM DCFH‐DA at 37°C in the dark for 30 min. Measurements were conducted promptly thereafter. To assess MMP, cells were stained using the Mitochondrial Staining Kit (JC‐1) labeled with FITC and PE (Multi Science, China; MJ101). Cells were treated with 2 µM JC‐1 dye and incubated at 37°C in a 5% CO_2_ atmosphere, protected from light, for 15 min. All flow cytometric analyses were carried out on a BD LSRFortessa cell analyzer (BD Biosciences, USA), and data processing was performed using FlowJo software.

### 4.7 Fluorescence Microscope

Confocal microscopy was employed to determine the localization of circRNA and UBQLN1. Immunofluorescence co‐localization was conducted following a previously established protocol. Briefly, cells were fixed in 4% paraformaldehyde for 20 min and subsequently rinsed with PBS. Permeabilization was performed using 0.2% Triton X‐100 in PBS for 10 min, followed by blocking with 10% goat serum. The anti‐UBQLN1 primary antibody (Abcam, UK; ab3341) was diluted 1:250 in the blocking buffer and applied to the cells for a 1‐h incubation at room temperature. After washing three times with PBS, cells were incubated with a goat anti‐rabbit IgG secondary antibody conjugated to Dylight649 (Multi Sciences, China; GAR6492) for 1 h at room temperature. Subsequently, the cells were treated with an anti‐DDDDK tag antibody (Abcam, UK; ab205606) for 1 h at room temperature, washed thoroughly, and then incubated with an Alexa Fluor 546‐labeled goat anti‐mouse IgG secondary antibody for another hour. Following final washes with PBS, nuclei were stained using DAPI (Beyotime, China; C1005) for 4 min. Imaging was performed using a Nikon A1 confocal microscope (Nikon, Japan). Additionally, circRNA was detected using a specific fluorescein‐conjugated probe.

### 4.8 Transmission Electron Microscope

Following the respective treatment, cells were harvested and fixed overnight at 4°C in 2.5% glutaraldehyde. Subsequent processing was carried out according to the protocols described in the relevant literature. Typically, after the initial fixation, cells were rinsed three times with 0.1 M PBS, followed by a secondary fixation in osmium tetroxide for 1 h. The samples were then subjected to dehydration through a graded ethanol series and subsequently embedded in acrylic resin. Ultrathin sections were stained using uranyl acetate and lead citrate before imaging. Transmission electron micrographs were acquired using an FEI Tecnai Spirit microscope operating at 120 kV.

### 4.9 Metabolite Content Detection

Intracellular levels of MDA were measured using a lipid peroxidation detection kit (Beyotime Biotechnology, China; S0131S). Cellular glutathione in its reduced (GSH) and oxidized (GSSG) forms was quantified employing GSH and GSSG assay kits (Beyotime Biotechnology, China; S0053). The ratio and content of NAD^+^/NADH were determined with the NAD^+^/NADH detection kit based on WST‐8 (Beyotime Biotechnology, China; S0175), while NADP^+^/NADPH levels were assessed using a similar WST‐8‐based detection kit (Beyotime Biotechnology, China; S0179). Intracellular glucose concentrations were evaluated by a glucose assay kit from Shanghai Cablebridge Biotechnology Co., Ltd. (China; MS2601). Pyruvate content was measured using the pyruvic acid assay kit (Shanghai Cablebridge Biotechnology Co., Ltd., China; MC8C7L), and lactate levels were assessed by the lactic acid assay kit (Shanghai Cablebridge Biotechnology Co., Ltd., China; MS2206). ATP concentrations inside the cells were quantified with an ATP detection kit (Beyotime Biotechnology, China; S0026). All assays were performed strictly following the manufacturers' protocols.

### 4.10 RNA Pull Down, RIP, and Co‐immunoprecipitation

Based on previous studies, RNA pull‐down, RIP, and co‐immunoprecipitation assays were conducted using HepG2‐SR cells. Cells were lysed in a buffer mixture combining western blot and immunoprecipitation (IP) buffers (Beyotime, China; P0013J), supplemented with protease inhibitors (Medchemexpress, USA; HY‐K0010) and RNase inhibitors (Sigma, USA; 3335399001). Dynabeads (Invitrogen, USA; 65801D), biotin‐labeled specific RNA probes, and the cell lysates were incubated together overnight at 4°C. For immunoprecipitation, Protein A/G Sepharose beads (Santa Cruz, USA; sc‐2003) were pre‐incubated overnight at 4°C with 5 µg of antibodies against rabbit IgG (Beyotime, China; A7016) or UBQLN1 (Cell Signaling Technology, USA; 14526), then combined with the lysates. Additionally, lysates from cells transfected with control, UBQLN1, or UBQLN1‐deletion plasmids tagged with FLAG were incubated overnight at 4°C with anti‐FLAG immunomagnetic beads. After incubation, beads were collected and subjected to multiple washes. Subsequently, proteins were extracted using RIPA buffer, and RNA was isolated with Trizol reagent.

### 4.11 HCC Mouse Model

A sorafenib‐resistant subcutaneous HCC mouse model was established using a patient‐derived xenograft approach. Fresh HCC tumor specimens from patients were initially implanted subcutaneously into the axillary region of NOD/SCID mice. After approximately 4 weeks, all mice began receiving sorafenib treatment. Following more than 12 weeks of continuous therapy, the enlarged xenografts were harvested and sectioned into smaller fragments. These tumor pieces were then transplanted into the axillae of 4‐week‐old male BALB/c nude mice. Upon the tumors reaching a diameter of around 3 mm, the nude mice were randomly allocated into two groups: one subjected to UBQLN1 knockdown and the other serving as a control. UBQLN1 expression was suppressed via siRNA transfection using cholesterol‐conjugated RIG‐I siRNA (RiboBio, China). Each tumor was locally injected with 5 nmol of siRNA every 3 days for a duration of 5 weeks. A similar procedure was employed to generate another cohort of nude mice, which were divided into GPX4 knockdown and control groups. Throughout the experimental period, all nude mice received sorafenib by oral gavage at a dosage of 30 mg/kg/day. Mice were euthanized after 5–6 weeks of treatment for subsequent analyses. The subcutaneous tumor size was recorded weekly using the formula: tumor volume (mm^3^) = (*L* × *W* ^2^)/2, where *L* denotes the longest diameter and *W* the shortest diameter.

NOD/SCID mice used in this study were purchased from Shanghai SLAC Laboratory Animal Co., Ltd. BALB/c nude mice were purchased from Hangzhou Qizhen Laboratory Animal Technology CO., Ltd. All mice were housed under specific pathogen‐free conditions. All animals were maintained in individually ventilated cages with a controlled environment, including a temperature of 22 ± 2°C and a relative humidity of 50 ± 10%. Mice had free access to standard rodent chow and sterile purified water. A 12‐h light/dark cycle was maintained throughout the study.

### 4.12 Statistical Analysis

Statistical analyses were performed using GraphPad Prism 8. To compare quantitative data between groups, either an unpaired or paired t‐test was applied as appropriate. Results are presented as the mean ± standard error of the mean based on a minimum of three independent experiments. A two‐tailed *p*‐value less than 0.05 was considered indicative of statistical significance in all analyses.

## Author Contributions

**Lin Ji**, **Junjie Xu**, and **Xiujun Cai** conceived and/or designed this study. **Lin Ji**, **Yeling Ruan**, **Meng Tong**, and **Tianyi Chen** performed experiments and analyzed data. **Jingwei Cai** and **Zhengtao Ye** processed the experimental data and performed statistical analysis. All authors contributed to interpreting the results. **Lin Ji**, **Yeling Ruan**, and **Tianyi Chen** drafted the manuscript, and all authors revised the manuscript. All authors approved the final version of the manuscript.

## Conflicts of Interest

The authors declare no conflicts of interest.

## Funding

This research was supported by Joint Funds of the National Natural Science Foundation of China under Grand No.U24A20762 (to Xiujun Cai), National Natural Science Foundation of China under Grant No.82471759 (to Junjie Xu) and No. 82271767 (to Junjie Xu), the Fundamental Research Funds for the Central Universities under No. 226‐2022‐00127 (to Junjie Xu), Natural Science Foundation of Zhejiang Province under Grant No. HDMD24H160007 (to Junjie Xu), Key Projects of the National Health Commission and Provincial Co‐construction Project under Grant No. WKJ‐ZJ‐2429 (to Junjie Xu).

## Ethics Statement

The study was approved by the Animal Ethics Committee of the Bioresources Center at the Science, Technology, and Research Institution of Sir Run‐Run Shaw Hospital, Zhejiang University School of Medicine (SRRSH2023‐0001).

## Consent

Written informed consent was obtained from all participants.

## Supporting information



Supporting Information
**FIGURE S1** circRNA‐SORE affects cellular reactive oxygen species (ROS) regulation and ferroptosis in SK‐Hep‐1 cells. (A) The RNA levels of circRNA‐SORE in parental and sorafenib‐resistant cells were examined via qRT‐PCR assays. (B) ROS levels were measured using a DCFH‐DA probe via flow cytometry in parental cells with or without circRNA overexpression for about 48 h. ROS levels were measured in sorafenib‐resistant cells with or without circRNA knockdown for 48 h. (C) Mitochondrial membrane potential levels were detected using the JC‐1 assay under the same conditions described in (B). (D) Representative transmission electron microscopy (TEM) images of mitochondrial morphological changes after circRNA knockdown for 48 h. Scale bar, 5 µm, 2 µm. (E) The size of mitochondria was quantified by ImageJ. (F) Representative TEM images of the changes in mitochondrial cristae after circRNA‐SORE depletion. Scale bar, 0.5 µm. (G) Cellular redox balances in sorafenib‐resistant cells with or without circRNA‐SORE depletion were monitored by detecting the level of redox pairs like NAD/NADH and NADPH/NADP. (H) ATP levels in sorafenib‐resistant cells with or without circRNA knockdown. *p < 0.05, **p < 0.01, ***p < 0.001, and ****p < 0.0001. ns. not statistically significant.
**FIGURE S2** UBQLN1 affected cellular reactive oxygen species (ROS) regulation and ferroptosis in SK‐Hep‐1 cells. (A) ROS levels were measured using a DCFH‐DA probe by flow cytometry with or without UBQLN1 knockdown in sorafenib‐resistant cells treated with sorafenib. (B) Representative transmission electron microscopy (TEM) images of mitochondrial morphological changes during UBQLN1 knockdown. Scale bar, 5 µm, 2 µm. (E) Mitochondrial area was measured by ImageJ.

## Data Availability

Datasets used and/or analyzed are available upon reasonable request to the corresponding author.
