# Supplementary material for: circRNA‐SORE/UBQLN1/GPX4 Mediates the Acquisition of Sorafenib Resistance in Hepatocellular Carcinoma Through Inhibition of Ferroptosis
Source: MedComm (2020). 2025 Nov 23;6(12):e70488. doi: 10.1002/mco2.70488 (PMC12640610; doi:10.1002/mco2.70488)
Supplement: Supplementary file 1 — Supporting Information FIGURE S1 circRNA‐SORE affects cellular reactive oxygen species (ROS) regulation and ferroptosis in SK‐Hep‐1 cells. (A) The RNA levels of circRNA‐SORE in parental and sorafenib‐resistant cells were examined via qRT‐PCR assays. (B) ROS levels were measured using a DCFH‐DA probe via flow cytometry in parental cells with or without circRNA overexpression for about 48 h. ROS levels were measured in sorafenib‐resistant cells with or without circRNA knockdown for 48 h. (C) Mitochondrial membrane potential levels were detected using the JC‐1 assay under the same conditions described in (B). (D) Representative transmission electron microscopy (TEM) images of mitochondrial morphological changes after circRNA knockdown for 48 h. Scale bar, 5 µm, 2 µm. (E) The size of mitochondria was quantified by ImageJ. (F) Representative TEM images of the changes in mitochondrial cristae after circRNA‐SORE depletion. Scale bar, 0.5 µm. (G) Cellular redox balances in sorafenib‐resistant cells with or without circRNA‐SORE depletion were monitored by detecting the level of redox pairs like NAD/NADH and NADPH/NADP. (H) ATP levels in sorafenib‐resistant cells with or without circRNA knockdown. *p < 0.05, **p < 0.01, ***p < 0.001, and ****p < 0.0001. ns. not statistically significant. FIGURE S2 UBQLN1 affected cellular reactive oxygen species (ROS) regulation and ferroptosis in SK‐Hep‐1 cells. (A) ROS levels were measured using a DCFH‐DA probe by flow cytometry with or without UBQLN1 knockdown in sorafenib‐resistant cells treated with sorafenib. (B) Representative transmission electron microscopy (TEM) images of mitochondrial morphological changes during UBQLN1 knockdown. Scale bar, 5 µm, 2 µm. (E) Mitochondrial area was measured by ImageJ. [file MCO2-6-e70488-s001.pdf]

Supplementary Information

circRNA-SORE/UBQLN1/GPX4 mediates the acquisition of sorafenib resistance in hepatocellular carcinoma through inhibition of ferroptosis

Lin Ji [1,2#], Yeling Ruan[2,3#], Meng Tong[1,2#], Tianyi Chen[2,4#], Jingwei Cai[2,5], Zhengtao Ye[1,2], Xiujun Cai[1,2]* and Junjie Xu[1,2]*

1 Department of General Surgery, Sir Run-Run Shaw Hospital, Zhejiang University School of Medicine, Hangzhou 310016, China.
2 National Engineering Research Center of Innovation and Application of Minimally Invasive Instruments, 310016, Hangzhou, China
3 Department of Head and Neck Surgery, Sir Run-Run Shaw Hospital, Zhejiang University School of Medicine, Hangzhou 310016, China.
4 Plastic & aesthetic center, Sir Run-Run Shaw Hospital, Zhejiang University School of Medicine, Hangzhou 310016, China.
5 Department of Thoracic Surgery, Sir Run-Run Shaw Hospital, Zhejiang University School of Medicine, Hangzhou 310016, China.
* Correspondence: walter235@zju.edu.cn (XJJ), srrsh_cxj@zju.edu.cn(CXJ)
\# These authors contributed equally to this work

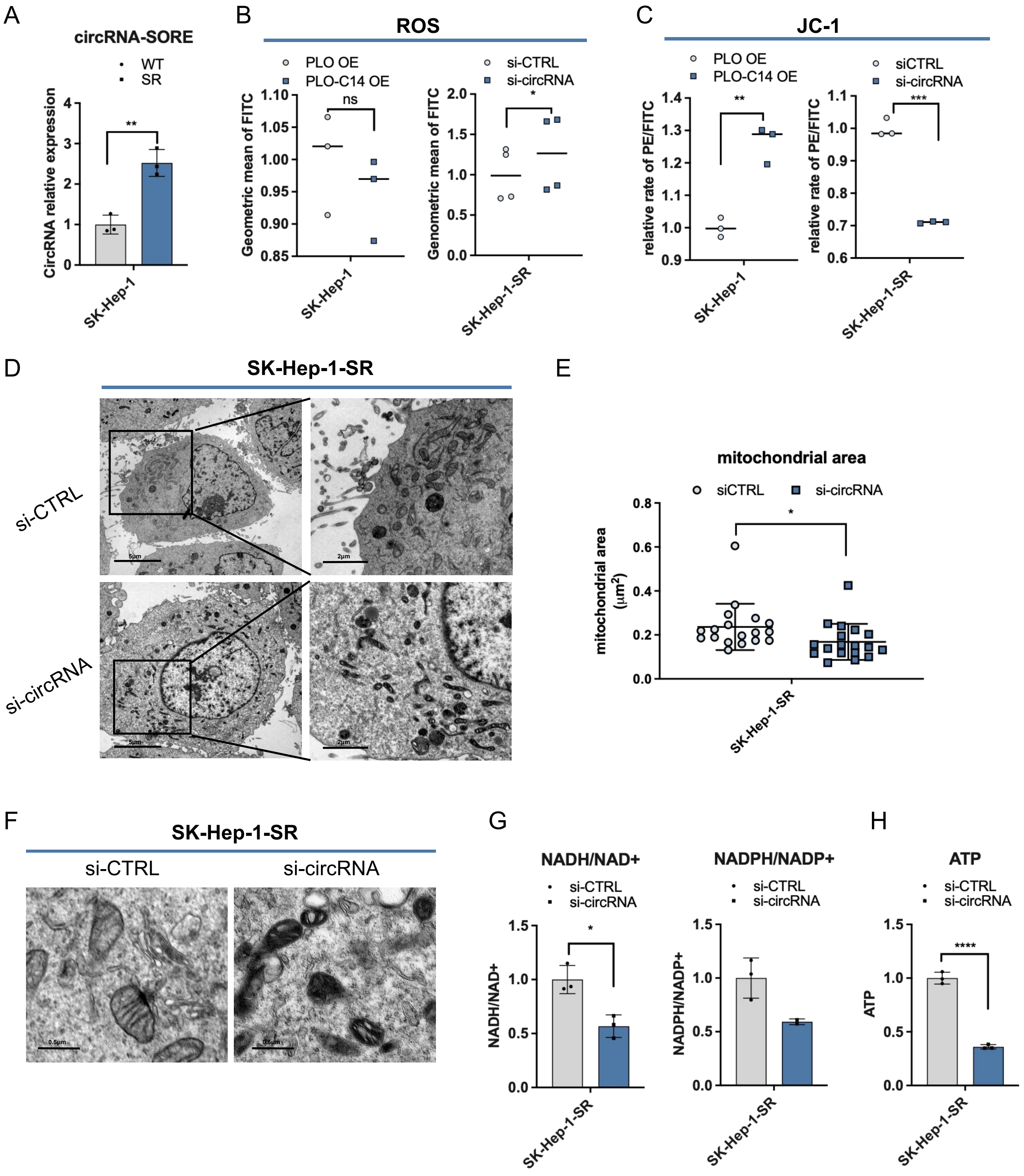
A
circRNA-SORE
WT
SR
CircRNA relative expression
**
SK-Hep-1
B
ROS
PLO OE
PLO-C14 OE
Geometric mean of FITC
ns
SK-Hep-1
si-CTRL
si-circRNA
Genometric mean of FITC
*
SK-Hep-1-SR
C
JC-1
PLO OE
PLO-C14 OE
relative rate of PE/FITC
**
SK-Hep-1
siCTRL
si-circRNA
relative rate of PE/FITC
***
SK-Hep-1-SR
D
SK-Hep-1-SR
si-CTRL
si-circRNA
5μm
2μm
E
mitochondrial area
siCTRL
si-circRNA
mitochondrial area (μm²)
*
SK-Hep-1-SR
F
SK-Hep-1-SR
si-CTRL
si-circRNA
0.5μm
G
NADH/NAD+
si-CTRL
si-circRNA
NADH/NAD+
*
SK-Hep-1-SR
NADPH/NADP+
si-CTRL
si-circRNA
NADPH/NADP+
SK-Hep-1-SR
H
ATP
si-CTRL
si-circRNA
ATP
****
SK-Hep-1-SR

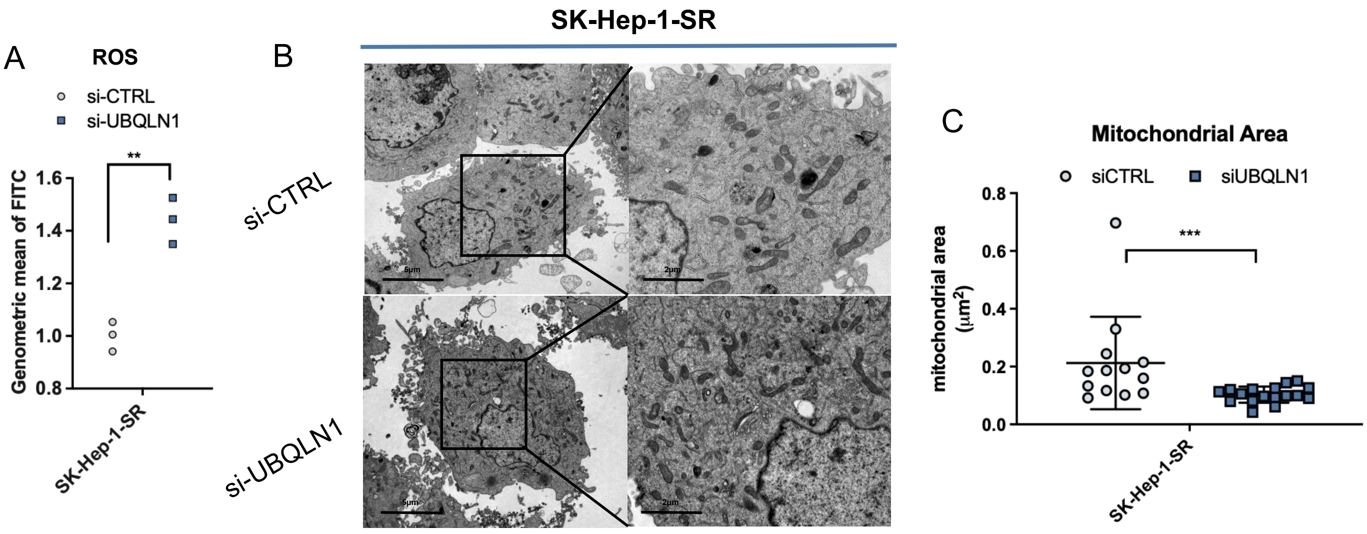
A
ROS
si-CTRL
si-UBQLN1
**
Genometric mean of FITC
1.6
1.4
1.2
1.0
0.8
SK-Hep-1-SR
B
SK-Hep-1-SR
si-CTRL
si-UBQLN1
C
Mitochondrial Area
siCTRL
siUBQLN1
***
mitochondrial area (μm²)
0.8
0.6
0.4
0.2
0.0
SK-Hep-1-SR

Figure S1: circRNA-SORE affects cellular ROS regulation and ferroptosis in SK-Hep-1 cells

(A)The RNA levels of circRNA-SORE in parental and sorafenib-resistant cells were examined via qRT-PCR assays. (B) ROS levels were measured using a DCFH-DA probe via flow cytometry in parental cells with or without circRNA overexpression for about 48 hrs. ROS levels were measured in sorafenib-resistant cells with or without circRNA knock down for 48 hrs. (C) Mitochondrial membrane potential levels were detected using JC-1 assay under the same condition described in B. (D) Representative TEM images of mitochondrial morphological changes after circRNA knockdown for 48h. Scale bar, 5 μm, 2 μm. (E) The size of mitochondria was quantified by ImageJ. (F) Representative TEM images of the changes in mitochondrial cristae after circRNA-SORE depletion. Scale bar, 0.5 μm. (G) Cellular redox balances in sorafenib-resistant cells with or without circRNA-SORE depletion were monitored by detecting the level of redox pairs like NAD/NADH and NADPH/NADP. (H) ATP levels in sorafenib-resistant cells with or without circRNA knockdown. *p < 0.05, **p < 0.01, ***p < 0.001, and ****p < 0.0001. ns. not statistically significant

Figure S2: UBQLN1 affected cellular ROS regulation and ferroptosis in SK-Hep-1 cells

(A) ROS levels were measured via using a DCFH-DA probe by flow cytometry with or without UBQLIN1 knockdown in sorafenib-resistant cells treated with sorafenib. (B) Representative TEM images of mitochondrial morphological changes during UBQLN1 knockdown. Scale bar, 5 μm, 2 μm. (E) Mitochondiral area was measured by ImageJ.
